# Identification of transcriptome induced in roots of maize seedlings at the late stage of waterlogging

**DOI:** 10.1186/1471-2229-10-189

**Published:** 2010-08-25

**Authors:** Xiling Zou, Yuanyuan Jiang, Lei Liu, Zuxin Zhang, Yonglian Zheng

**Affiliations:** 1 National Key Laboratory of Crop Genetic Improvement, Huazhong Agricultural University, Wuhan, 430070, China

## Abstract

**Background:**

Plants respond to low oxygen stress, particularly that caused by waterlogging, by altering transcription and translation. Previous studies have mostly focused on revealing the mechanism of the response at the early stage, and there is limited information about the transcriptional profile of genes in maize roots at the late stage of waterlogging. The genetic basis of waterlogging tolerance is largely unknown. In this study, the transcriptome at the late stage of waterlogging was assayed in root cells of the tolerant inbred line HZ32, using suppression subtractive hybridization (SSH). A forward SSH library using RNA populations from four time points (12 h, 16 h, 20 h and 24 h) after waterlogging treatment was constructed to reveal up-regulated genes, and transcriptional and linkage data was integrated to identify candidate genes for waterlogging tolerance.

**Results:**

Reverse Northern analysis of a set of 768 cDNA clones from the SSH library revealed a large number of genes were up-regulated by waterlogging. A total of 465 ESTs were assembled into 296 unigenes. Bioinformatic analysis revealed that the genes were involved in complex pathways, such as signal transduction, protein degradation, ion transport, carbon and amino acid metabolism, and transcriptional and translational regulation, and might play important roles at the late stage of the response to waterlogging. A significant number of unigenes were of unknown function. Approximately 67% of the unigenes could be aligned on the maize genome and 63 of them were co-located within reported QTLs.

**Conclusion:**

The late response to waterlogging in maize roots involves a broad spectrum of genes, which are mainly associated with two response processes: defense at the early stage and adaption at the late stage. Signal transduction plays a key role in activating genes related to the tolerance mechanism for survival during prolonged waterlogging. The crosstalk between carbon and amino acid metabolism reveals that amino acid metabolism performs two main roles at the late stage: the regulation of cytoplasmic pH and energy supply through breakdown of the carbon skeleton.

## Background

Waterlogging, caused by flooding, long periods of rain, and poor drainage, is a serious abiotic stress determining crop productivity worldwide [1]. Depletion of oxygen is a major feature of waterlogging, because the diffusion of oxygen in water is 10^-4 ^times slower than that in air [2]. The imbalance between slow diffusion and rapid consumption of oxygen in plant roots drastically reduces the oxygen supply [3], which is vital to the survival of plant roots.

Plants respond to low oxygen through specific alterations of transcription and translation. The response was first studied in maize roots. Using two-dimensional electrophoresis, about 20 anaerobic proteins (ANPs) were shown to be induced during low oxygen treatment, while synthesis of aerobic proteins was drastically repressed [4]. Most of the ANPs were identified as enzymes involved in sugar phosphate metabolism, such as *alcohol dehydrogenase*, *aldolase, enolase*, *glucose phosphate isomerase*, *glyceraldehyde-3-phosphate dehydrogenase*, *pyruvate decarboxylase *and *sucrose synthase *[5,6]. Studies have subsequently identified alterations in the expressions of hundreds of genes in response to oxygen deficiency. These genes can be grouped into four subsets. Firstly, sensing and cell signaling, involving transient induction of mitochondria *alternative oxidase *(AOX) [7,8] and activation of *RopGAP4 (Rop *GTPase activating protein4) [9,10], which is related to the ROS species signaling pathway. The induction of *calmodulin *[11] and *CAP *(*Calmodulin*-*Associated Peptide*) is verified to have an important role in Ca^2+ ^signaling [12,13]. In addition, various plant growth regulators involved in signaling cascades influencing cellular response are also induced under waterlogging condition [7,14-25]. Secondly, metabolic adjustment, reflected as a switch from aerobic respiration to anaerobic fermentation involving induction of ANPs acting in sugar phosphate metabolism, such as lactic and alcoholic fermentation. Thirdly, maintenance of pH; in addition to the role of ANPs related to metabolic switch [5,26,27], the activation of plant *glutamate decarboxylases *(*GADs*) interacting with calmodulin is related to pH regulation [7,28-30]. Lastly, other proteins, which include non-symbiotic hemoglobin, a protein that has been reported to be associated with many biological systems in hypoxia response [31-34]. Nitrogen metabolism [35] and cell wall loosening [36] were also identified to participate in the response to oxygen depletion.

Waterlogging can be conceptually divided into three time stages. The first stage (0-4 h) consists of the rapid induction of signal transduction components. This initial signal reception response in turn activates the second stage (4-24 h), a metabolic adaptation, including the induction of glycolytic and nitrogen metabolic pathways. In the third stage (24-48 h), which involves the formation of aerenchyma and the induction of xyloglucan endotransglycosylase, programmed cell death occurs in the roots [14].

Recently, Qiu *et al*. (2007) identified 34 QTLs for waterlogging tolerance in a set of F_2:3 _families derived from HZ32 (tolerant inbred) × K12 (sensitive inbred) [37]. Several major QTLs for waterlogging tolerance were mapped on chromosomes 4 and 9. Secondary QTLs influencing tolerance were located on chromosomes 1, 2, 3, 6, 7 and 10.

Although many studies of the molecular mechanism of tolerance to waterlogging have been reported, there have been relatively few studies of transcriptomic and proteomic changes in maize [6,38-40] compared with rice [41], Arabidopsis [7,42,43], and other species [44]. In addition, these transcriptomic and proteomic studies mostly focused on the response to waterlogging at the early stage (0-8 h) in roots of maize seedlings. The late response (after 12 h) of gene expression in root cells of maize is unknown. To gain an insight into gene expression changes in response to waterlogging at the late stage, a forward SSH library from four time points (12 h, 16 h, 20 h and 24 h) after waterlogging treatment was constructed using the tolerant inbred line HZ32. A total of 296 unigenes were identified as being induced by waterlogging and were clustered into 13 categories based on Gene Ontology analysis, suggesting the response involved a broad spectrum of genes and complex biological pathways. In addition, 63 unigenes were identified to be co-located with QTLs for waterlogging tolerance by an in silico mapping approach, and are thus important candidates for further breeding of waterlogging-tolerant crops.

## Methods

### Plant material and growth conditions

Seeds of HZ32 (waterlogging tolerant inbred line) were germinated for three days and the seedlings were individually transplanted into sand chambers. Plants were grown under 30°C:22°C (light:dark, 16:8 h) until they initiated three leaves in total with two leaves expanded. Uniform seedlings were selected and divided into two groups. One group was cultured with a normal water supply as the control and the other was submerged in water with all leaves in air as the treatment.

### RNA isolation

Roots treated for different time periods (12 h, 16 h, 20 h and 24 h) were immediately harvested, with eight seedlings representing a sample, and stored at -70°C. Roots of the controls were also harvested at the corresponding time point.

Total RNA was isolated using TRIzol (Invitrogen, USA) following the manufacture's recommendations. RNA quantity and quality were assessed by a Nanodrop spectrophotometer (Nanodrop Technologies, Montchanin, DE) and by agarose gel electrophoresis.

### Construction of the SSH library

For the library, equal amounts (250 μg) of total RNA from the four time points were pooled to form the mRNA (2 μg) tester, while mRNA generated from the roots under normal conditions was used as the driver. SSH was performed with a PCR-select cDNA subtraction kit (Clontech, Japan) according to the manufacturer's instructions. Subtracted PCR amplified cDNAs were cloned into a pGEM-T vector (Promega, USA).

### Differential screening using reverse northern blotting

To screen the true-positive clones, the inserts of all clones in the library were amplified by PCR with nested primers NP1 and NP2R. Approximately 5 μL of PCR product was mixed with 5 μL of 0.6N NaOH for denaturing, and 1.5 μL of each sample was then arrayed onto two identical Hybond-N^+ ^nylon membranes (Amersham, UK). Membranes were neutralized in 0.5 M Tris-HCl (pH 7.5) for 2 min, and rinsed in distilled water for 30 s. They were cross-linked by baking for 2 h at 80°C and then stored at -20°C for later use.

Equal amounts (5 μg) of total RNA from four time points were pooled, with treatment and control respectively, and reverse transcribed using M-MLV RTase cDNA Synthesis Kit (Takara, Japan) in presence of α-[^32^P] dCTP and used as probes. Membranes were hybridized at 65°C with radio-labeled probes in hybridization buffer (Toyobo, Japan) for 14 h, then washed with 2× SSC, 0.5% (w/v) SDS at 65°C for 10 min, and 0.1 × SSC, 0.1% SDS at 25°C for 10 min. Membranes were exposed to X-ray film (Fuji photo film, Japan) at -80°C for signal detection. Reverse Northern blotting was performed twice with a biological replication of the probes. The signals of the corresponding clones from both hybridizations were compared and the positive clones identified in both replications were selected. The randomly selected positive clones were sequenced (Invitrogen, USA).

### Sequence analysis

Nucleotide sequences of ESTs > 100 bp were analyzed. Adaptor and vector sequences were removed with Phred-Phrap analysis software. All overlapping sequences were clustered into singleton contigs using DNAstar. A similarity search was performed by basic local alignment search tool (BLAST) at National Centre for Biotechnology Information (NCBI, NIH, USA) http://www.ncbi.nlm.nih.gov/BLAST/. In particular, blastn and blastx analysis were performed with an inclusion score > 50. Functional classification of ESTs was carried out according to the functional categories of Gene Ontology (GO) http://www.geneontology.org.

### Real-time quantitative RT-PCR

Three biological replications of total RNA were used for real-time quantitative RT-PCR. Total RNA was treated with RNase-free DNase. Reverse transcription of total RNA (5 μg) was performed with an M-MLV RTase cDNA Synthesis Kit (Takara, Japan) following the manufacturer's instructions. Gene specific primers were designed using PRIMER3 software http://www.ncbi.nlm.nih.gov/tools/primer-blast/ (Table 1). For each gene amplified, 0.5 μl of cDNA was used for the detection of amplified products.

**Table 1:** Gene-specific primers for real time PCR

Primer name	Primer
*isovaleryl-CoA dehydrogenase*	CCACCACTACACTATCCCCTC
	AGCGAACCAAACCACCGGCT
*prolyl 4-hydroxylase*	TTCCGCGCCCGCGAAGAAAA
	CAGGTGGGTGGTGTGGGGGA
*glutamate synthase1*	CGTGGTGGTTGTGGAGCGGA
	AAGCCCTTGCTTGCTGGCCC
*acetyl-coenzyme A carboxylase*	CGTGGTGGTTGTGGAGCGGA
	AAGCCCTTGCTTGCTGGCCC
*ethylene-responsive protein*	ATACGTCTCCGCCGCGTCCT
	GCGTGAGCTCCCTCTGCTGC
*alanine aminotransferase*	AAGGCCGAAGGAGCAATGTAT
	GCAGCCTCGATTGCCTTCT
*aspartate aminotransferase*	GGGTTGGATTTTGAGGGAAT
	CCACTGTTCAGGAGTTGGGT
*alcohol dehydrogenase*	GGGGATTCTGAAACCTGGAC
	ACCCTTCTCAGAACAACCCC
*sucrose synthase*	GGGGATTCTGAAACCTGGAC
	ACCCTTCTCAGAACAACCCC
*auxin response factor*	CACCAGGCACATGCAAAGAG
	TTTTCCTGGGAATGCTGTTTCT
*coproporphyrinogen III oxidase *	AAGGGGGAGGCCGGCAAGAA
	TACCCGCGGCGAAGAATGGC
*hypoxia induced protein*	GGTTGCTGGCTGCCTGGCTT
	CCGTACGACGCTGGCTCACG
*DNA-binding protein*	ACCGGGATTCCCTCCGCCAA
	TCACCGCCAGCTTGGCATCG
*ubiquitin-activating enzyme E1 *	ACCCGGGTGCACCATGGATCT
	CATCGCCACTAGCCGCTCCC
*putative MAPK *	TGCTGCAGCTGTGGACACCC
	CCCGCAGTATGCGTGTGGCA
*translation initiation factor eIF-2B*	CCCCTACCCCATCTCCCGGC
	CCTCCACCTTCCGGCGCTTG
*protein phosphatase 2A regulatory subunit A*	GCTCCTACCCTGCCCCGTCT
	GGGGTCCGGATCGGGCTTCT
*NADP-dependent malic enzyme*	AGCCAGTGGAGCCTTGGCCT
	GGAGCAAGTGGAGGCTGCCG
*Unigene285 *	TCGACAGCGGAGCGATCGAA
	CCCCCGGGCAGCACATACCT
*Unigene2*	ACCAGGCTGGGGCAAGAGGA
	ACCGTCACCACAGCGGGATCA
*pyruvate kinase*	CAGGCTTCCGTCACAGGCAGT
	CCAAGGCTCAAGAGATCACAGTTA
*organic anion transporter*	CGAGCCGTGACCACCCAACA
	GAAGATCTAGGCGGCGGCAGCA
*actin*	AAGTACCCGATTGAGCATGG
	GATGGAGTTGTACGTGGCCT

Selected gene transcripts were quantified by real-time PCR using SYBRGreen PCR Master Mix (Takara, Japan) with three replications. The expression of *actin *was used as a control. PCR amplification conditions were as follows: 95°C for 2 min, then 38 cycles of 95°C for 10 s, 60°C for 30 s and 72°C for 15 s. The reactions were carried out using a CFX96 Real-Time System C1000 Thermal Cycler (Bio-RAD, USA). The relative expressions of genes were normalized to *actin *expression in each set of samples, according to the manufacturer's instructions, and the results were analyzed by matched software.

### Physical mapping of waterlogging tolerance loci and integration of genetic mapping with induced genes

Waterlogging loci were identified using an F_2:3 _population from a cross between "HZ32" and "K12" using SSR markers [37], and the linked molecular markers were directly assigned to respective bacterial artificial chromosome (BAC) clones of maize inbred line B73 by searching in MaizeGDB http://www.maizegdb.org/. Subsequently, a physical map spanning the waterlogging tolerance loci was constructed in silico, based on the contig map.

The unigene sequences were assigned to the corresponding bacterial artificial chromosome (BAC) of maize inbred B73 using blastn, with an inclusion score > 100 and E-value < 10^-5^, and then compared with the physical map of waterlogging tolerance loci to identify the co-localized unigenes.

## Results

### Construction of the forward subtractive cDNA library and identification of up-regulated genes

To identify genes induced at the late stage after waterlogging, a forward subtractive cDNA library was constructed. To gain an indication of the subtraction efficiency, γ-*tubulin *transcript abundance (primers: γ-*tubulin *-F TCTCCAGGGTCCTCCATTCC, γ-*tubulin*-R TGTCGTCCAACCTTACAACTCACT) was compared before and after subtraction by PCR. The abundance of this non-differentially expressed gene was estimated to be ~2^6 ^fold less in the subtracted cDNA populations (data not shown).

In the screening of clones in the SSH library, a replication of the reverse northern blot was carried out, with a biological replication of RNA samples, and the results were consistent for more than 98% of the clones, except for some variations of quantity.

**Figure 1:**
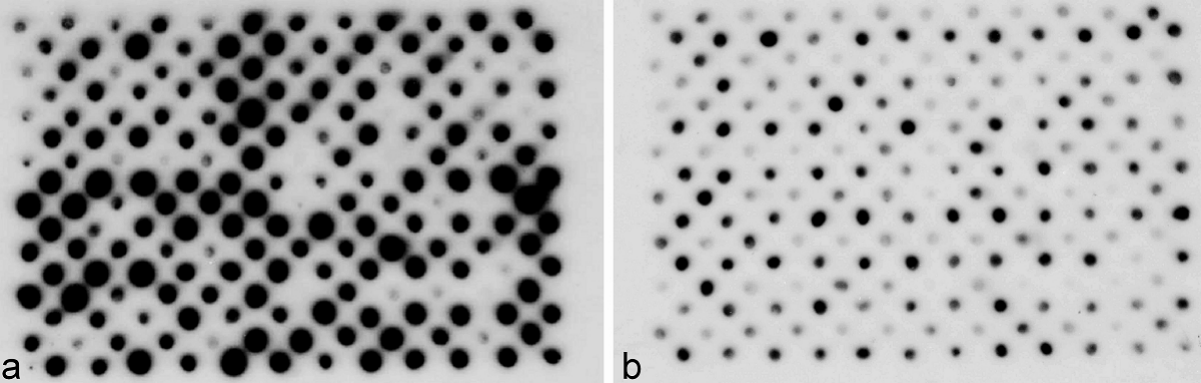
**Screening by reverse Northern**. One set of membranes is shown as an example. Each cDNA obtained from the SSH library was spotted onto a membrane. Membrane (a) and (b) were two identical membranes. Each blot was hybridized with ^32^P-labeled probes derived from 20 μg of total RNA. Membrane A was hybridized with total RNA from waterlogging-treated samples as a probe, and B was hybridized with total RNA from the control as a probe.

A total of 471 of positive clones (Figure 1) were randomly chosen and sequenced. 465 ESTs (expressed sequence tags) longer than 100 bp were obtained. The sizes of the original cDNA fragments ranged from 104 to 1081 bp, with an average size of approximately 332 bp.

### Annotation and functional cataloging of up-regulated genes

**Figure 2:**
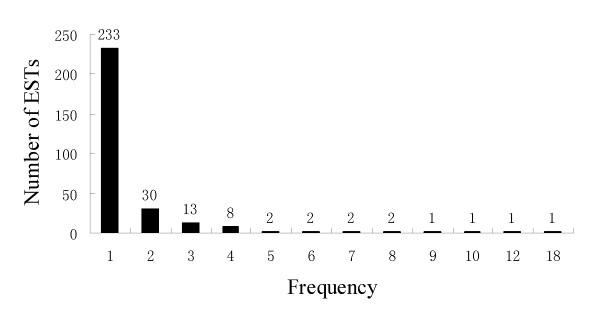
**ESTs redundancy in the SSH library**. Number of ESTs in each category is also presented at the top of each bar.

A total of 296 unigenes were assembled from the 465 ESTs, of which 233 were singletons and 163 were contigs represented by 2-18 overlapping ESTs (Figure 2). The most abundant unigene comprised 18 ESTs and was annotated as *nucleotide binding protein*, followed by a unigene comprising 12 overlapped ESTs encoding a universal stress protein. The third most abundant unigene encoded alanine aminotransferase and comprised 10 ESTs (Additional file 1).

**Figure 3:**
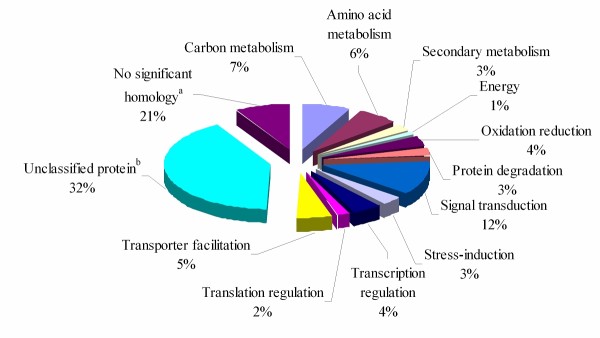
**Functional categorization of induced genes**. All 296 induced unigenes were functionally annotated using blastn and blastx at NCBI, and categorized based on GO annotation. Percentages of unigenes in each category are also presented at the top of each bar. ^a^These unigenes had no significant hits (score < 50) in blastn and blastx searches of GenBank. ^b^These unigenes matched proteins annotated as 'unknown protein' or 'hypothetical protein'.

The annotation of 296 unigenes in GenBank indicated that 8.4% (25/296) of them had no homology in the database. The average length of these unigenes with no homology was significantly shorter than the length of those with hits in the database (209 bp compared to 344 bp). Subsequently, the 296 unigenes were assigned into 13 functional categories by a Gene Ontology assay (Figure 3). Although almost half (49.3%) of the unigenes were clustered into "no significant homology" or "unclassified protein" categories, of the remainder, the main effects of waterlogging stress on cellular function at the late stage were signal transduction (11.8% of categorized genes), carbon metabolism (7.1% of categorized genes), and amino acid metabolism (6.4% of categorized genes). Additionally, genes encoding transporter facilitation comprised 5.4%. The remaining categorized unigenes (20.0%) were assigned to oxidation-reduction, protein degradation, stress-induction, translation regulation, energy, secondary metabolism, and transcription regulation.

Unlike signal cascade-associated genes identified at the early stage of waterlogging stress, signal cascade-associated genes detected at the late stress stage can be classified into three significant subgroups; genes catalyzing reversible phosphorylation, hormone responsive genes, and genes involved in small molecule related pathways. For reversible phosphorylation, genes induced by waterlogging include *lammer-type protein kinase*, *protein kinase APK1A*, *shaggy-related protein kinase*, *Mitogen-activated protein kinase *(*MAPK*), *protein phosphatase 2A*, *protein phosphatase 2C*, and *diacylglycerol kinase*. The hormone signal-associated genes up-regulated by waterlogging mostly encode plant hormone response factors, such as *auxin response factor*, *coronatine-1-insensitive protein 1*, and *ethylene-responsive protein*. Small molecule signal transduction processes have been shown to have a well-established role under waterlogging stress. Several genes encoding small signal molecules were up-regulated at the late stage of waterlogging stress. Two genes encoding, respectively, calcium ion binding protein and calreticulin, which are known to be responsible for the balance of Ca^2+ ^signal transduction, were identified in the forward library, indicating that the Ca^2+ ^mediated signal cascade is potentially modulated by waterlogging in root cells. As well as genes encoding small GTP binding proteins, guanine nucleotide-binding protein, ADP-ribosylation factor 1, and ras-related protein were identified in the library, implying that the GTPase rheostat is involved in regulation of transcription at the late stage. A number of other genes that might be involved in signal transduction in response to waterlogging at the late stage were also found, including *amelogenin precursor like protein*, *WD-repeat protein-like*, *calpain-like protein*, and *TBC domain containing protein*.

16% unigenes (48/296) that were included in the category "metabolism" can also be further clustered into three subgroups, carbon metabolism, amino acid metabolism, and secondary metabolism. For carbon metabolism, several genes encoding ANPs were identified, including *sucrose synthase 1*, *glucose-6-phosphate 1-dehydrogenase*, *glyceroldehyde-3-phosphate dehydrogenase*, *pyruvate kinase*, and *alcohol dehydrogenase*, which are involved in multiple steps in glycolysis and fermentation pathways, which is consistent with results from previous studies in Arabidopsis and maize [4,45]. A gene encoding glucose-6-phosphate 1-dehydrogenase (G6PDH, EC 1.1.1.49) was induced under waterlogging stress. G6PDH controls the flux through a non-reversible limb of the oxidative pentose phosphate pathway and is responsible for generation of NADPH. Malic enzyme was also up-regulated and encodes a proton-consuming enzyme involved in a potential mechanism of preventing pH decline [5,26]. Nineteen unigenes encoding enzymes involved in amino acid metabolism, such as *alanine aminotransferase*, *aspartate aminotransferase*, *isovaleryl-CoA dehydrogenase*, *ferredoxin-dependent glutamate synthase*, and *prolyl 4-hydroxylase alpha-2 *were identified. It is interesting to note that an abundant unigene in this category was *Alanine aminotransferase *(comprising 12 ESTs), which showed significant induction by waterlogging stress. The category "secondary metabolism" comprised eight unigenes (3%), encoding enzymes such as sirohydrochlorin ferrochelatase, isopentenyl-diphosphate delta-isomerase II, and dihydroflavonol-4-reductase. In this category, the gene encoding coproporphyrinogen III oxidase [EC 1.3.3.3] was also obtained in our forward SSH library, which catalyses oxidative decarboxylation of coproporphyrinogen III to proto-porphyrinogen IX in the heme.

### Verification of SSH data by real-time quantitative RT-PCR

**Figure 4:**
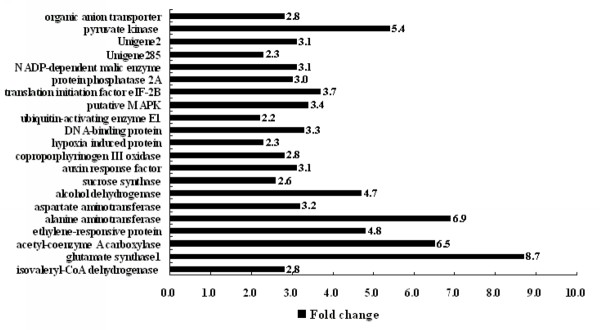
**Verification of SSH results by real time PCR**. The transcriptional levels of candidate genes was examined by real time PCR with three biological replications of pooled RNA. The fold change is the ratio of the expression of genes in the treatment compared to the control.

To validate the results of the SSH library, the transcriptional level of 21 unigenes were examined by real time PCR using the same RNA for the library and two other biological replication of the RNA. Consistent with reverse northern blot, these unigenes exhibited > 2 fold higher expression in response to waterlogging (Figure 4). However samples of *prolyl 4-hydroxylase alpha-2 subunit *did not yield a signal above the detection threshold, which revealed that the transcripts of this gene are present at about a 2^-10 ^lower level than the housekeeping gene (Actin) (data not shown).

**Figure 5:**
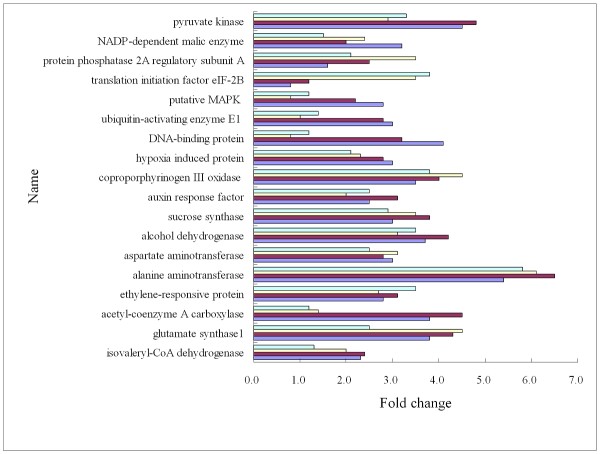
**Verification of SSH results by real time PCR at each time point**. The level of transcriptional of candidate genes was examined by real time PCR with three biological replications at each time point. The fold change is the ratio of the expression of genes in the treatment compared to the control.

Additionally, real time PCR was performed for 19 of the 21 unigenes at each time point used in SSH library construction. The results (Figure 5) show that not every gene showed increased expression at each time point. For example, genes encoding acetyl-coenzyme A carboxylase, DNA-binding protein, ubiquitin-activating enzyme E1, and putative MAPK did not change significantly at 12 h and 16 h, and showed increased expression at the two later time points. The gene encoding translation initiation factor eIF-2B showed increased expression at 12 h and 16 h, and decreased to a normal level at 20 h and 24 h. Other genes showed increased expression at all four time points.

### Integration of up-regulated genes with mapped QTLs

QTLs for waterlogging tolerance were mapped to the B73 physical map [37]. This allowed us to determine whether the genes induced in the tolerant line HZ32 were located in the QTL regions.

**Figure 6:**
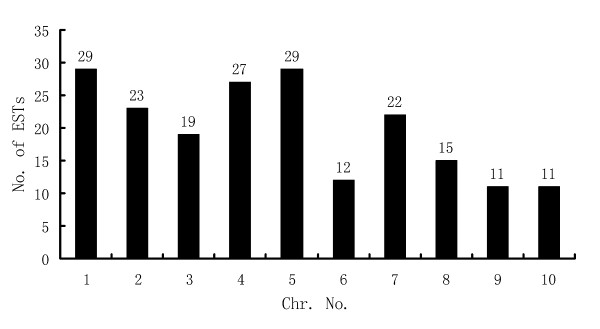
**Distribution of induced unigenes on chromosomes**. Numbers of unigenes on each chromosome are also presented at the top of each bar.

Of the 296 unigenes, 198 were mapped to the maize physical map using blastn. The chromosomal locations of these genes are shown in Figure 6. Chromosomes 1 and 5 had the most ESTs (29 ESTs on each chromosome); chromosome 9 and 10 had the least (11 ESTs only, respectively).

**Figure 7:**
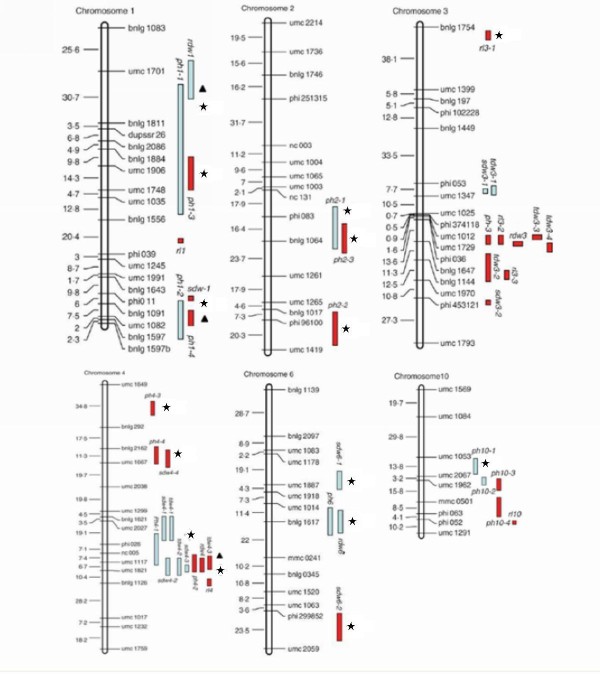
**Distribution of co-localized unigenes on maize chromosomes**. The panes indicate the regions covered by QTLs identified by Qiu (2007); the black stars indicate that multiple ESTs were assigned to single loci; the black triangles indicate that single ESTs were assigned to single loci.

63 (21.1%) unigenes were in silico mapped to genomic regions harboring multi-trait or single-trait QTLs (Figure 7). A total of 21 (33.3%) unigenes have no functional annotation, representing hypothetical proteins. Another 42 unigenes were genes related to various pathways, such as *alanine aminotransferase*, *ADP-ribosylation factor*, *glucose-6-phosphate 1-dehydrogenase*, *acetyl-coenzyme A carboxylase*, and *alcohol dehydrogenase2 *(Table 2). The co-localized unigenes were distributed on chromosomes 1, 2, 3, 4, 6, and 10, and more than half (60%) were on chromosomes 1 and 4.

**Table 2:** QTL information for co-localized genes in the forward SSH library

Chr. No.	Unigene	**ID**^**a**^	**QTL**^**b**^	**Annotation**^**c**^
1	278	HO068217	Ph1-1	calpain-like protein
1	245	HO068192	Ph1-1	protein kinase C inhibitor
1	118	HO068078	Ph1-1	WD-repeat protein-like
1	281	HO068220	Ph1-1	40 S ribosomal protein
1	78	HO068280	Ph1-1	ADP-ribosylation factor 1
1	292	HO068229	Ph1-1	pyruvate dehydrogenase E1
1	160	HO068116	Ph1-1	26 S proteasome regulatory particle non-ATPase subunit5
1	22	HO068177	Ph1-1/Ph1-3	vacuolar protein sorting 26
1	1	HO068150	Ph1-1/Ph1-3	alanine aminotransferase 2
1	10	HO068069	Ph1-1/Ph1-3	alanine aminotransferase 2
1	210	HO068162	Ph1-1/Ph1-3	wound responsive protein
1	115	HO068075	Ph1-1/Ph1-3	putative glutathione peroxidase
1	116	HO068076	Ph1-1/Ph1-3	putative glutathione peroxidase
1	13	HO068100	Ph1-1/Ph1-3	hypothetical protein
1	102	HO068062	Ph1-1/Ph1-3	succinyl-CoA ligase alpha-chain 2
1	100	HO068060	Ph1-1/Ph1-3	vacuolar ATP synthase
1	52	HO068258	Ph1-1/Ph1-3	hypothetical protein
1	34	HO068239	Ph1-1/rdw1	putative cystatin
1	215	HO068166	Ph1-2/ph1-4	tryptophanyl-tRNA synthetase
1	139	HO068099	sdw-1	hypothetical protein
1	140	HO068101	sdw-1	hypothetical protein
2	102	HO068062	Ph2-1	succinyl-CoA ligase alpha-chain 2
2	25	HO068205	Ph2-1	sirohydrochlorin ferrochelatase
2	198	HO068147	Ph2-1/Ph2-3	60 S ribosomal protein
2	23	HO068187	Ph2-1/Ph2-3	topoisomerase-like protein
2	145	HO068106	Ph2-2	pyruvate kinase
2	137	HO068097	Ph2-2	hypothetical protein
2	138	HO068098	Ph2-2	hypothetical protein
2	171	HO068125	Ph2-2	hypothetical protein
2	117	HO068077	Ph2-2	tRNA-splicing endonuclease subunit
3	15	HO068115	Rl3-1	hypothetical protein
3	164	HO068119	Rl3-1	NADP-dependent malic enzyme
4	50	HO068256	Ph4-1/sdw4-1/tdw4-1	CAAX prenyl protease 1
4	194	HO068144	Ph4-1/sdw4-1/tdw4-1	hypothetical protein
4	176	HO068128	Ph4-1/sdw4-1/tdw4-1	glyceroldehyde-3-phosphate dehydrogenase GAPC3
4	135	HO068095	Ph4-1/sdw4-1/tdw4-1	CAAX prenyl protease 1
4	136	HO068096	Ph4-1/sdw4-1/tdw4-1	CAAX prenyl protease 1
4	8	HO068291	Ph4-1/sdw4-1/tdw4-1/sdw4-2/tdw4-2/ph4-2/tdw4-3	CAAX prenyl protease 1
4	232	HO068180	Ph4-3	pollen signalling protein with adenylyl cyclase activity
4	247	HO068194	Ph4-3	pollen signalling protein with adenylyl cyclase activity
4	7	HO068281	Ph4-3	pollen signalling protein with adenylyl cyclase activity
4	162	HO068118	Ph4-3	hypothetical protein
4	244	HO068191	Ph4-3	CAND1 binding (CAND1)
4	78	HO068280	Ph4-4/sdw4-4	ADP-ribosylation factor 1
4	202	HO068153	Ph4-4/sdw4-4	hypothetical protein
4	9	HO068295	Ph4-4/sdw4-4	hypothetical protein
4	219	HO068168	Ph4-4/sdw4-4	nucleotide binding protein
4	17	HO068131	Sdw4-3/sdw4-2/tdw4-2/ph4-2/tdw4-3	alcohol dehydrogenase2
4	236	HO068184	Sdw4-3/sdw4-2/tdw4-2/ph4-2/tdw4-3	hypothetical protein
4	177	HO068129	Sdw4-3/sdw4-2/tdw4-2/ph4-2/tdw4-3	hypothetical protein
6	207	HO068158	ph6/rdw6/Sdw6-2	hypothetical protein
6	287	HO068224	ph6/rdw6/Sdw6-2	CCR4-NOT transcription complex subunit 6
6	129	HO068088	ph6/rdw6/Sdw6-2	hypothetical protein
6	178	HO068130	Sdw6-1/Sdw6-2	hypothetical protein
6	109	HO068068	Sdw6-1/Sdw6-2	vacuolar ATP synthase subunit B
6	110	HO068070	Sdw6-1/Sdw6-2	vacuolar ATP synthase subunit B
6	230	HO068178	Sdw6-2	hypothetical protein
6	161	HO068117	Sdw6-2	transport protein particle subunit
6	96	HO068293	Sdw6-2	MPK17-1 - putative MAPK
10	13	HO068100	Ph10-1	hypothetical protein
10	234	HO068182	Ph10-1	glucose-6-phosphate 1-dehydrogenase
10	141	HO068102	Ph10-1	acetyl-coenzyme A carboxylase
10	72	HO068275	Ph10-1	hypothetical protein

**^a ^**Accession numbers of unigenes.

**^b ^**The QTLs were identified by *Qiu*, and sdw = shoot dry weight; ph = plant height; rl = root length; rdw = root dry weight; tdw = total dry weight. For all the QTL names, the first number following the letters represents the chromosome locations of the QTL and the second number represents the orders of the QTL located on the same chromosome by the same trait.****The sequence was searched in MaizeGDB using blastn of an inclusion score >100 and E-value < 10^-5^.

**^c ^**The annotation was obtained by a similarity search using blastn and blastx at NCBI.

In the study of QTLs for waterlogging tolerance, many regions were identified by more than one QTL. As a result, 33 ESTs (52.4%) were mapped to regions identified by more than one QTL (2-7 QTLs).

## Discussion

### The reliability of the SSH library

At each step in the production of SSH data, variability can be introduced leading to potential errors. To control against negative results due to individual differences, samples were pooled from eight seedlings. To limit false positive genes, two biological replications of RNA from four time points were used to screen induced genes in the library by reverse northern blotting. The results showed that although there was some biological variation, the data interpretation was not affected. The results were further confirmed by real time PCR experiments for 21 selected waterlogging-responsive genes with three biological replications of the RNA, including the same samples of RNA used in the SSH experiment and reverse northern blotting. The expression analysis of selected genes indicated that all were induced by waterlogging, as detected in the library, except for one gene with extremely low expression. The results of real time PCR performed at each time point revealed that not every gene was up-regulated during the entire late stage, which suggested that, although the expression profiles of genes identified in the SSH library were complicated, all the genes identified in the SSH library was induced at least one of the four time points.

### Crosstalk between various pathways

**Figure 8:**
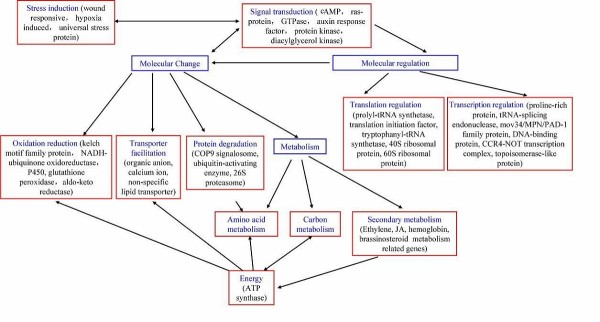
**Crosstalk between the categories of induced genes**. Red frames represent categories with functional annotation; blue characters for names of categories and black ones for genes; the bidirectional arrow represents the presence of crosstalk.

The response to waterlogging is always via a network, and crosstalk takes place between different pathways. This idea was supported by the identification of genes classified into different categories at the late stage of waterlogging. Although the genes identified were categorized into nine categories by functional annotation, there was no clear boundary among these categories. Based on the results revealed by genes obtained from the SSH library, some possible links between the categories can be deduced (Figure 8).

### Signal transduction events and crosstalk among various pathways

Signal cascades occurring in maize roots subjected to waterlogging provide significant clues for understanding the regulation of gene expression. In this study, similar to the results revealed by both Klok and Liu in Arabidopsis under hypoxia stress [7,42], genes related to various signal cascades identified in the SSH library were overrepresented at the late stage. Although genes involved in signal transduction were also found in the previous study at the early stage (1-8 h), they were completely different [38-40]. These genes mainly act in three different signal pathways. For example, a gene encoding putative mitogen-activated protein kinase (MAPK) was up-regulated under waterlogging. MAPK is a signal molecule mediating protein phosphorylation directly or indirectly [46]. Plant MAPK cascades have been implicated in the development and responses to stress. In Arabidopsis, stress-responsive MAP kinase (MAPK) 6 leads to phosphorylation of ACC synthase (ACS) [47], which is related with accumulation of ethylene, a well known molecule in the response to waterlogging [48]. It was reported that the formation of aerenchyma in roots, the key response for survival of prolonged waterlogging after 24 h, was triggered by ethylene. Furthermore, there was a breakthrough in the study of plant adaptation to anaerobiosis, which was closely related to ethylene. Map-based cloning and characterization of two major QTLs for tolerance of flooding in rice revealed that both Snorkel [49-51] and Sub 1A [25,52] represented genes encoding ethylene-responsive factor-type transcription factors and are connected to gibberellin biosynthesis or signal transduction. However, the two genes regulated different mechanisms of adaptation to flooding; escape by elongation or remaining stunted under water until the flood recedes [53]. Thus, the results suggested that induction of MAPK in roots subjected to waterlogging could promote a MAPK cascade to regulate ethylene biosynthesis, which in turn regulates plant hormone cascades, finally resulting in modulation of morphological adaptation.

A gene encoding coproporphyrinogen III oxidase [EC 1.3.3.3] was obtained in our forward SSH library, which catalyses oxidative decarboxylation of coproporphyrinogen III to proto-porphyrinogen IX in the heme. Previous studies suggested that hemoglobin was up-regulated under hypoxia in barley, maize roots, and embryos [54] and has an important role in survival at the late stage of waterlogging. Over-expression of hemoglobin in maize could actually enhance root growth [55], as was found in other species [56,57]. Hemoglobin may serve as an O_2 _sensor [58], or act as an O_2 _carrier, as in animal cells in stressed tissues [59], to preserve oxygen-obligated metabolism, such as ethylene synthesis, in plants. At this late stage, synthesis of hemoglobin could be an important step in activating the next stage in the response to waterlogging.

A gene encoding prolyl 4-hydroxylase (P4H) [1.14.11.2], another oxygen sensor [60], was up-regulated in maize roots under waterlogging. P4 H has been proven to control a transcription factor (hypoxia-inducible heterodimeric transcription factor, HIF), a global regulator of hypoxia, through proline hydroxylation in various mammals [61]. Prolyl 4-hydroxylase targets proline residues of HIF for rapid ubiquitination and proteasomal degradation when oxygen is available. HIF is accumulated when oxygen is limited [62,63]. However, there has been little evidence of a role of P4 H in the regulation of the hypoxia response by modulating the expression of specific transcription factors in plants [64]. A recent study reported that overexpression of *AtP4H1 *in Arabidopsis could mediate and mimic the low oxygen response [65]. In maize, the P4 H gene has not been cloned, possibly due to its low level of expression. The advantage of the SSH technique for accumulating low abundance transcripts has resulted in the identification of a P4 H transcript in our library. The induction of *P4 H *under waterlogging stress implied the possible participation of the P4 H signaling pathway at the late stage of waterlogging stress.

Although the two induced genes discussed above were categorized in "transporter activity" or "amino acid metabolism", they are also related to signaling events. This implies that signal transduction is not linear, but that crosstalk exists among different biochemical pathways, such as hemoglobin and P4 H.

Previous studies have reported that signal transduction events mostly occur at the early stage of waterlogging [14]. To our surprise, many (more than 11%) of the induced genes obtained in the library were involved in various signaling pathways. This suggested that the response to waterlogging at the late stage was more complicated than was expected. It was reported that the third stage (24-48 h) was important for survival of prolonged exposures to low oxygen tension, and involves the formation of aerenchyma in the roots [5], which was not a direct consequence of oxygen deficiency, but was presumably triggered by stage 1 and/or stage 2 genes [66].

We propose that the response to waterlogging in the roots of maize seedlings occurs in two phases (defense and adaptation). At the early stage, it is most important that plants can sense the lack of oxygen around the root system and trigger initial changes to gene expression, while at the late stage, signal transduction pathways different from the early stage should be activated to regulate the tolerance mechanism for survival under prolonged waterlogging.

### Crosstalk between carbon utilization and amino acid metabolism

Carbon utilization is a well-known mechanism regulated under waterlogging. Many studies revealed that genes responsible for carbon utilization were induced in response to hypoxia in many species. Consistent with results from previous expression profiling and proteomic studies in rice [41,67] and Arabidopsis [7,42,43,68], a number of genes involved in the glycolysis pathway were identified, implying that glycolysis is still active at the late stage under waterlogging, for supply of carbon source and energy [66,69,70]. In theory, breakdown of carbohydrates is the main pathway of carbon flux and energy supply. Actually, intermediates from lipid and protein degradation also act as important carbon sources entering glycolysis under waterlogging.

Many genes encoding proteins or enzymes involved in degradation of lipids and proteins were identified in our SSH library, which supports the hypothesis mentioned above. Although genes involved in protein degradation were identified at the early stage [38-40], it is interesting that more than 3% of the genes induced by waterlogging were related with protein degradation, such as *COP9 signalosome complex subunit 3*, *ubiquitin-conjugating enzyme*, *ubiquitin-activating enzyme E1*, and *26 S proteasome regulatory particle non-ATPase*. The results implied that degradation of aerobic proteins would help decrease the consumption of oxygen, and abundant free amino acids should be derived at the same time. Free amino acids have two destinations, protein synthesis and amino metabolism. Hypoxia can repress overall protein synthesis in plants [4]. Accordingly, accumulated free amino acids from protein degradation should act as substrates in amino acid metabolism.

Importantly, the gene encoding Alanine aminotransferase (AlaAT; E.C. 2.6.1.2), which is involved in amino acid metabolism, was induced in root cells of maize seedlings under waterlogging conditions. AlaAT catalyzes reversible transfer of an amino group from alanine to 2-oxoglutarate to form pyruvate and glutamate. The induction of *AlaAT *under hypoxia, accompanied by the accumulation of alanine, has been reported in barley [71,72], rice [73], soybean [74], and Arabidopsis [7,75]. In Arabidopsis, a study on an AlaAT1 mutant (*alaat1-1*) demonstrated that AlaAT primarily catalyzes the breakdown of alanine under hypoxia stress, instead of synthesis of alanine [76]. In other words, there are other pathways for the accumulation of alanine that do not include the AlaAT pathway.

The gene encoding ferredoxin-dependent glutamate synthase (GS) was also up-regulated in response to waterlogging at the late stage. GS is involved in the GS/GOGAT cycle, which is used to store carbon and nitrogen, and is responsible for regenerating glutamate [77]. Glutamate has been shown to be a critical product for regulation of cytoplasmic pH. It is catalyzed by glutamate decarboxylase (GAD; EC 4.1.1.1 [EC] 5), consuming a proton and synthesizing GABA [30,78,79]. GABA can be converted to succinate and enter carbon metabolism. This metabolic pathway involves GABA-T and SSADH, and this route for glutamate carbon to enter the tricarboxylic acid cycle (TCA cycle) is called the GABA shunt. Ala is synthesized from pyruvate in this process, which suggests the GABA shunt is responsible for accumulation of Ala under waterlogging conditions.

Considering of the information above, it is possible that Ala can be accumulated from protein degradation and the GABA shunt, and that there is a cycle between pyruvate and Ala. In this study, a gene encoding aspartate aminotransferase was shown to be induced under waterlogging, which can generate Glu and OAA from Asp and 2-oxoglutarate. OAA can be converted to malate, which has a critical role in regulation of cytoplasmic pH, catalyzed by malic enzyme, which was also upregulated in this study.

**Figure 9:**
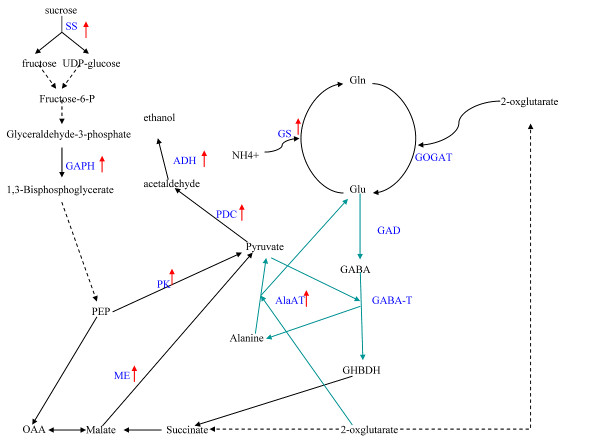
**The crosstalk between carbon and amino acid metabolism**. Black characters represent products in the pathway; blue characters represent enzymes involved in the pathway; the red arrow represents the gene that was identified to be induced in our library.

It seems that in root cells of maize seedlings at the late stage of waterlogging stress, amino acid metabolism has two main roles (Figure 9), a) generating Glu and Ala, which are critical for regulation of cytoplasmic pH; and b) break down of carbon skeletons and generating intermediates for energy metabolism.

Recently, most studies of carbon unitilization for flooding tolerance response focused on *pdc *and *adh *in this complicated cycle of pathways. It was not surprising that phenotypic analysis of over-expression of *pdc *or *adh *in rice [14,80,81], arabidopis [82], cotton [83], and other species has provided different conclusions. Based on the results revealed this study, genes involved in protein degradation and amino acid synthesis are ideal candidate genes that enhance flooding tolerance of plants along with *pdc/adh*.

### Mapping differentially expressed genes in the response to waterlogging

For this and other studies, the ultimate aim is to clone the genes responsible for tolerance to waterlogging. The classic approach is map-based cloning. However, the development of molecular markers and genotyping a mapping population is very expensive and time consuming. On the other hand, a large number of genes involved in the response to waterlogging can be obtained using the technique of SSH. The candidate gene approach is thus more convenient and cost saving. Localized on a QTL linkage map, they may be seen as positional candidate genes for further investigation by functional analysis.

Therefore, the latter strategy was adopted to analyze all 296 up-regulated unigenes. Only 63 (21.1%) unigenes were located in the regions identified by QTLs. There could be two reasons for this. First, the genome sequence of maize is not perfect and contains many gaps http://www.maizesequence.org, and significant differences of sequence exist among in maize inbred lines [84-86]. These could be responsible for the failure of up to 33% of the unigenes to be localized on the map. Second, the fact that an EST was not co-located with a QTL does not imply that the EST was not important for waterlogging tolerance, because the lack of functional polymorphism between parental alleles of a gene can result in the absence of a QTL.

The co-located unigenes should be further verified before in-depth study. Due to the limited mapping resolution used in the previous study, the positions of these identified QTLs could not be accurately determined, which can cause false positives for the candidate gene approach. Further detailed genetic analysis is needed to determine whether the genes in these regions represent candidate genes for these QTLs. Nevertheless, this is the first step in combining expression profiles and QTL mapping in the study of the tolerance of waterlogging. It is complementary to map based cloning and could used for marker-assisted selection in breeding.

## Conclusion

To gain an insight into how the roots of maize seedlings respond to waterlogging at the late stage, we carried out gene expression profiling at four time points (12 h, 16 h, 20 h, and 24 h) after waterlogging treatment using tolerant inbred line HZ32. Annotation and analysis based on gene ontology terms suggested that waterlogging affected a broad spectrum of functional categories.

At the late stage of waterlogging, amino acid metabolism plays an important role related to protein degradation and carbon metabolism. It is thought to be involved in regulation of cytoplasmic pH and breakdown of carbon skeletons for the supply of energy. Signal transduction is still active and is different from those signaling pathways induced in early stages, possibly because of the need to regulate the tolerance mechanism for survival under prolonged waterlogging. We propose that the response to waterlogging should be conceptually divided into two stages: defense and adaption. The new genes related to signal transduction identified in this study might perform key roles in regulating the response to waterlogging at the late stage and provide new insights into the response to waterlogging in maize.

A total of 63 candidate genes for waterlogging tolerance were validated by *in silico *mapping through a candidate gene approach. These genes might be important candidates for further breeding of waterlogging-tolerant crops, but will require further verification.

The identification of specific genes affecting complex traits is always one of the most difficult tasks in genetics. By studying which genes are induced at the late stage in response to waterlogging in the roots of maize seedlings, we have provided the basis for further investigation in this field. Sense/antisense over-expression of these genes in transgenic plants could be used to identify their contribution to waterlogging tolerance.

## Authors' contributions

YZ conceived of the study and participated in its design. ZZ was involved in designing and planning the work, and in interpreting the results. XZ carried out most of the work and drafted the manuscript. YJ participated the screening of the library and real time PCR analysis. LL helped with the electronic mapping. All authors have read and approved the final manuscript.

## Supplementary Material

Additional file 1 **Redundancy, annotation and functional categories of unigenes in the SSH library**. ^**a **^The IDs of sequences submitted to GenBank. ^**b **^The length of contigs. ^**c **^ESTs longer than 100 bp were assembled using DNAstar. ^**d **^Functional classification of unigenes was carried out according to functional categories of Gene Ontology (GO) http://www.geneontology.org. ^**e **^The IDs of sequences with homology in GenBank. ^**f **^Annotation analysis was based on blastn and blastx at NCBI with an inclusion score > 50. ^**g **^The max score based on blastn and blastx at NCBI. Click here for file
